# Antibiotic prescribing practices in paediatric septic shock in a tertiary care hospital in a resource limited setting: an audit

**DOI:** 10.11604/pamj.2019.34.133.15820

**Published:** 2019-11-07

**Authors:** Varsha Vekaria-Hirani, Rashmi Kumar, Rachel Musoke

**Affiliations:** 1 Paediatric Department, MP Shah Hospital, Nairobi, Kenya; 2 Paediatric Department, University of Nairobi, Nairobi, Kenya

**Keywords:** Septic shock, antibiotics, paediatrics, resource limited setting, Kenya

## Abstract

**Introduction:**

Early empiric broad spectrum antibiotic administration in children with septic shock improves outcome. Knowledge on possible bacterial aetiology, drug resistance pattern and rational choice of antibiotics is crucial in management of septic shock.

**Methods:**

This was an audit carried out among 50 (0- 5 years age) children admitted with septic shock at the Kenyatta National Hospital between October to December 2016. A standard questionnaire was used for data collection as per the Surviving Sepsis Guideline. Data were stored in Excel and analyzed in Strata 12.

**Results:**

Of the 50 admitted children with septic shock 86% were less than one-year age. Samples for blood cultures were removed from 12(24%) prior to administration of antibiotics. Blood culture bottles were unavailable in 80%. All children received antibiotics. Antibiotics were initiated in 44(88%) in the golden hour of diagnosis of septic shock. Monotherapy with cephalosporins 30 (60%) was the commonest choice of initial antibiotic. Antibiotics were changed in 7(22.6%) and 1(5.3%) at 24 and 48 hours respectively due to clinical deterioration. Over mortality at 72 hours was 35 (70%). All the 9 children initiated on meropenem monotherapy on admission died.

**Conclusion:**

The majority of patients with septic shock were under one-year age. All patients were initiated on antibiotics. Blood cultures were done in a quarter of the patients. Monotherapy with cephalosporin was the commonest choice of antibiotic. De-escalation was not well accomplished due to microbiological culture limitation. There was no standard antibiotic choice hence antibiotic use in septic shock needs to be included in the paediatric local guidelines.

## Introduction

Sepsis, a life-threatening organ dysfunction syndrome, is caused by an uncontrolled host response to infection, whereas, septic shock is a subset of sepsis with profound circulatory, cellular and metabolic abnormalities associated with a greater risk of mortality than with sepsis alone [1]. Clinical signs needed to recognize septic shock include signs of suspected sepsis, systemic inflammatory response syndrome (SIRS) and altered tissue perfusion. As infection causes sepsis, managing infection is one of the most critical components of sepsis therapy [2]. Among the numerous major advances in the revision of surviving sepsis guidelines, antibiotic therapy was one of the domains in which the most important changes and advances were made [3]. Early empiric broad-spectrum antibiotic therapy is of paramount importance towards a better outcome, as a direct correlation exists between delayed initiation of antibiotics and risk of organ dysfunction [4,5]. Though early initiation of empirical antibiotic treatment in critical illnesses is crucial, it remains challenging due to the various factors that need to be considered, like the most probable pathogen, right drug or combination of antibiotics, any organ impairment, with a focus on achievement of high concentrations of antibiotics in the affected organ, minimal side effects and eliminating the infectious process towards better patient outcomes [6]. The empiric choice of antibiotics in severe sepsis/shock should be based on the likely source of infection, age of the child, knowledge on disease prevalence, drug resistance patterns, recent antibiotic use (within previous three months) and a previous documentation of some specific colonized or infecting bacteria [7,8]. Organisms differ by local geography, age and medical co-morbidities, overcrowding and poor vaccination coverage increasing the risk of infection and septic shock [9]. In the paediatric population, positive culture yields in septic shock can be as low as 18% [10,11]. In neonates presenting with septic shock, the most common bacteria include coagulase negative Staphylococcus, Group B Streptococci, Escherichia coli, Candida and Anaerobes. Group B Streptococci infection can progress to septic shock in more than 50% neonates infected with this pathogen [12]. In young children, common causative organisms include Streptococcus pneumoniae, haemophilus influenzae type b and neisseria meningitides, staphylococcus aureus, streptococcus pyogenes and candida [13]. Viral-bacterial co-infection occurs in up to 34% of cases of severe pneumonia, resulting in a higher likelihood of respiratory failure and septic shock [14]. Immunosuppressed children carry a higher risk of invasive infections with multi-organ involvement through various pathogens [15,16].

Suitable initial antimicrobial therapy is defined as treatment with an antibiotic regime, known to have a confirmed in vitro action against the identified bacterial species associated with infection [17]. The impact of choice of antibiotics is most apparent on patients who are severely sick. Therapeutic drug monitoring in children to ensure target levels and avoid drug toxicity and source control strategies such as drainage or debridement of infected tissues and removal of infected devices or foreign bodies have an important role in optimal management [18]. In neonatal sepsis, use of ampicillin and gentamicin is recommended for early onset neonatal sepsis (≥72 hours). In late onset neonatal sepsis (>72 hours), similar broad-spectrum antibiotics are used as in early onset sepsis but 3^rd^ generation cephalosporins such as cefotaxime are added. Vancomycin is used instead of ampicillin in catheter-associated infections [19]. In children aged >1 month, a broad-spectrum antibiotic that covers both gram positive and gram-negative organisms are recommended, but the antibiotic choice depends on the clinical presentation, for example pneumonia, blood stream infection, intra-abdominal sepsis or meningitis that caused septic shock. International guidelines such as surviving sepsis guidelines 2012 recommends antibiotic administration within 1 hour of recognition of septic shock and a delay in administration is associated with high mortality rates [9]. Clear evidence states that in the management of patients with severe infections, not only should the antibiotic be appropriate, but initiation should also be prompt. Kumar *et al* in 2006 observed a strong correlation between delays in initiating antibiotic therapy and in-patient mortality in 2154 patients with septic shock; the time to initiation of appropriate antibiotic therapy being the strongest predictor of survival. The survival rate was noted to be 79.9% if effective antibiotics were administered within 1^st^-hour of documented hypotension, mortality increasing by a rate of 7.6% with each hour delay, in the initial 6 hours [20]. The identified multiple reasons like delay in initiation of antibiotics in severe infections underscores a systematic approach in the management of patients with serious infections. Iregui *et al* in 2002 noted that a delay in initiation of antibiotics for ≥24 hours led to a significantly high hospital mortality rate of 69.7% [21]. As observed in many studies, infection with a resistant pathogen is associated with a delayed initiation of appropriate antibiotics [22]. In a 3-year retrospective analysis of patients with MRSA infection, initiation of appropriate antibiotics within the first 24 hours after collection of culture-positive specimen was correlated with a higher survival rate [23].

Though adequate antimicrobial therapy is crucial for survival in patients with severe infections, inadequate therapy significantly increases the length of hospitalization in critically ill patients with severe sepsis or septic shock [24,25]. But without microbiological information, empirical therapy forms the backbone of therapy in critically ill patients. De-escalation is reduction in the spectrum of administered antibiotics through discontinuation of antibiotics providing activity against non-pathogenic organisms, discontinuation of antibiotics with similar activity or switching to an agent with narrower spectrum. To reduce antibiotic related side effects, extra costs and emergence of antibiotic resistance, de-escalation is a recommended approach with data suggesting improved outcomes in critically ill patients with severe sepsis [26]. At 48-72 hours, depending on the clinical status, response to management and microbiological data, appropriateness of the antibiotic therapy is evaluated and therapy is changed or narrowed as indicated [27]. If evidence points away from bacterial infection, de-escalation can be extended to discontinuation of antibiotic therapy [28]. International guidelines recommend the use of broad-spectrum antibiotics in patients with severe sepsis and septic shock, to minimize the risk of inadequate treatment of critically ill patients, but this has shown to increase mortality if used irrationally [7,29]. Resource-limited set-ups lack proper evidence and guidelines to monitor empiric antibiotic decisions for management of critically ill children. No studies have been done locally on use of antibiotics in critically ill children in septic shock and there were no local guidelines at the time of the study. Monitoring of local antibiotic prescribing practices with respect to the diagnosis is crucial to improve the prescribing practices. Judicious and apt empiric antibiotics can improve outcomes in critically ill patients with infection. Audit of antibiotic prescription practices provide the basis of development of specific antibiotic prescribing guidelines, which contribute to appropriate use of antibiotics. Audit of antibiotic use in septic shock, a common presentation of critically ill children in the outpatient department in resource limited set-ups, will improve our care for these children, correct errors and improve gaps in knowledge on antibiotic use among children. The information obtained from the study will guide us on staff training, making septic shock antibiotics management guidelines and policies for the hospital on appropriate use of antibiotics.

## Methods

This was an audit carried out over a 3-month period (October to December 2016) at the Kenyatta National Hospital (KNH). KNH is an 1800 bedded tertiary care hospital, the national teaching and referral hospital. Children aged 0 days to 12 years are admitted to the paediatric section of the hospital. The objectives were to audit the antibiotic prescribing practices among children aged 0 days to 5 years with septic shock, admitted at the Kenyatta National Hospital paediatric wards, newborn unit and paediatric intensive care unit. Of the 194 children aged 0 days (term neonate ≥37 weeks) to 12 years, with suspected sepsis screened during the study period from the paediatric emergency unit, 50 children were clinically diagnosed with septic shock. A child was diagnosed to have septic shock when he/she had clinical signs of SIRS and all signs of abnormal perfusion (capillary refill time >2 seconds, cold extremities, weak or absent radial pulse and altered mental status). Antibiotic practices were audited in these 50 children. Children with birth asphyxia, trauma, burns, anaphylaxis, liver failure, known cardiac disease, chronic renal failure, diarrhoea and severe acute malnutrition were excluded. Ethics approval was obtained from KNH/UON-ethics committee (P228/03/2016). Informed consent was obtained from the participant's parent/guardian to get enrolled in to the study. An audit on the antibiotic practices was carried out from the recruited patient's file and treatment sheets at 1st hour of recognition of shock, 24 and 48 hours and outcome recorded at 72 hours. All children received other care of septic shock as per the surviving sepsis 2012 Guideline. The data was then filled in a pretested questionnaire. The number of children who died during the study was recorded. Data was stored in MS-EXCEL and analysed using STATA. Frequency and percentages were calculated for categorical variables.

## Results

Of a total of 194 children aged 0 days to 5 years with suspected sepsis, the 50 children diagnosed with septic shock were enrolled in to the study. The socio-demographic characteristics of the population are as shown in Table 1. Empiric antibiotics were initiated in all 50 children admitted with septic shock on admission. A large majority, 49 (98%) of the patients received appropriate dose of the antibiotics for their age, based on body-weight or age-based formulae calculated dose. Samples for blood culture were removed from 12 (24%) patients prior to antibiotic administration and not done in 38 (76%) patients. The reason for not undertaking blood cultures was failed orders by the attending clinician in 20% and non-availability of blood culture bottles in 80% cases. A positive yield was not recorded from any of the samples taken. Types of antibiotics used were diverse. Monotherapy was the commonest approach of antibiotic prescription. Only a minority of the population studied received combination therapy. Mortality at 72 hours was 35(70%) and half dying in the first 24 hours. Case fatality versus choice of antibiotic revealed a high percentage of poor outcomes among children who received monotherapy with meropenem as shown in Table 2. Antibiotics were initiated in 44 (88%) of the study population within the first one hour of diagnosis of septic shock and 6 (12%) of the patients received antibiotics after one hour of diagnosis of septic shock, though none were initiated on antibiotics within the first 5 minutes as recommended by the surviving sepsis 2012 guideline. In 7(22.6%) of 31 alive and 1(5.26%) of 19 alive children, antibiotics were changed at 24 hours and 48 hours respectively based on documentation of clinical deterioration (worsening of respiratory rate, heart rate, deteriorating consciousness or perfusion). The antibiotic change was from third generation cephalosporins to carbapenems at 24 hours, except in one patient where the change to carbapenem was at 48 hours. Blood culture results did not yield any growth, hence antibiotic change was dependent on clinical deterioration only.

**Table 1:** Socio-demographic characteristics of enrolled children diagnosed with septic shock

Variable	Characteristic	Enrolled children with septic shock N=50 (%)
Age (Months)	< 1	20 (40)
	1 – 11	23 (46)
	12 – 59	7 (14)
Sex	Female	32 (64)
	Male	18 (36)
Referred from Another Hospital	No	12 (24)
	Yes	38 (76)

**Table 2:** Audit on antibiotic use in septic shock on admission

Antibiotic used		Administered on admission n=50 (%)	Case fatality at 72 hours (%)
**Monotherapy**			
Ceftriaxone		11(22)	8(72.7)
Ceftazidime		11(22)	7(63.6)
Meropenem		9(18)	9(100)
**Combination therapy**			
Crystalline penicillin and gentamycin		3(6)	3(100)
Ceftriaxone and	Amikacin	1(2)	1(100)
	flucloxacillin	3(6)	0(0)
Ceftazidime and	Amikacin	2(4)	1(50)
	Flucloxacillin	1(2)	0(0)
Meropenem and	Vancomycin	5(10)	4(80)
	Flucloxacillin	2(4)	0(0)
	Amikacin	2(4)	2(100)

## Discussion

The majority of our patients diagnosed with septic shock were infants and neonates, comparable to studies showing that neonates, infants and younger children are at increased risk for severe infections compared to older children and adults [30]. There was no significant gender difference in the population diagnosed with septic shock. Of note is that a great number (76%) of our critically-ill enrolled patients were referred in from other health facilities, increasing the possibility of infection with resistant organisms, frequent antibiotic resistance being a common scenario in low and middle-income countries [23]. All patients in the study population, n=50 (100%), were initiated on empirical antibiotics, conforming to various studies and Surviving Sepsis Guidelines stating intravenous antimicrobial therapy remains one of the strategic treatments in patients with severe sepsis and septic shock [1,7,31]. Also, majority of the study population received appropriate dose of antibiotics, though age-based formulae had to be used occasionally for dosing due to lack of consistent availability of a functioning calibrated weighing scale. Implementation of locally appropriate guidelines and training to improve care of seriously ill children through Emergency Triage and Assessment Treatment plus (ETAT +) could have played a major role in assisting a slow but steady improvement in the management of critically ill children [32]. But, to ensure the optimal antimicrobial treatment in a resource limited set-up like ours can be challenging due to the wide range of infectious diseases and causative microorganisms including bacterial, fungal and parasitic pathogens [33]. Culture of microbiological samples could not be conducted as required due to resource limitations and of the few samples cultured, all had a negative yield. As sensitivities of microbiological specimens is low even in developed countries, sensitivities of microbiological cultures in resource-limited settings is expected to be even lower due to lack of appropriate resources, culture media and inoculation methods [34]. Inadequate submissions and insufficient amounts of samples and prior antibiotic use could have resulted in a negative yield from the culture specimens in our study [35,36].

Monotherapy was the commonest approach of antibiotic prescription in our study, n=31 (62%). Bajcetic *et al*. recommend avoidance of combination therapy, with priority to monotherapy and consider it to be as efficient as combined therapy [6]. But a widely accepted fact leading to the utilization of initial combination therapy in septic shock clinical guidelines is an increased spectrum of coverage, allowing a higher probability of appropriate antimicrobial therapy [7]. Also, a reduced risk of emergence of resistance during therapy and a potential synergistic effect leading to more rapid pathogen clearance have been considered as a potential advantage of combination therapy over monotherapy [37,38]. But various studies over the years have found no significant mortality benefit with combination therapy [39-41]. Cochrane reviews performed by Paul and colleagues in 2014 failed to demonstrate improvement of outcome with combination therapy [42]. Several observational studies have suggested benefit with empirical combination therapy in high-risk but not low-risk patients. A weak recommendation was made for the use of empirical combination therapy in patients with septic shock (but not in sepsis without shock). This recommendation was based on the increasing frequency of antibiotic resistance, but to reduce inadequate coverage of pathogens in the critically ill septic shock children using combination therapy [3]. Majority of our patients were initiated on intravenous antibiotics within the initial one hour of diagnosis of septic shock. A major retrospective analysis of septic shock suggested that a delay in the early initiation of antimicrobial therapy is the single most critical determinant of survival, observed only 50% of patients received appropriate treatment during the 1st 6 hours with the median time noted to be 6 hours to initiate appropriate antimicrobial therapy [20]. Additional retrospective studies have confirmed that mortality in septic conditions including septic shock is increased with significant delays in antimicrobial administration [43].

Third generation cephalosporins were the most commonly prescribed empirical therapy in our study (Ceftriaxone and Ceftazidime). This antibiotic choice is similar to studies done in India and Pakistan on the empirical choice of antibiotics for very sick children [44,45]. Community and hospital-acquired infections with klebsiella, acinetobacter and pseudomonas have demonstrated 61.1%-85.6% resistance to Ceftriaxone and Cefotaxime [46]. Enterobacteriaceae also demonstrated similar trends with resistance to ampicillin 79.6%, gentamicin 22.2% and ceftriaxone 74.1% in Asian countries, studies from Africa also demonstrated high to moderate levels of resistance to ampicillin and gentamicin at 92.9% and 42.9% respectively. Ceftriaxone resistance to klebsiella spp. was also noted to be high in Africa at 50.0%. Le Doare *et al* in 2014 also highlighted that approximately 75% of isolates in Africa are multi-drug resistant (MDR) [46]. In our study, antibiotics were changed in a few patients after 24 hours and 48 hours. Reasons for antibiotic change in the study subjects being persistence of signs and symptoms of septic shock and clinical deterioration. Relevant microbiological data are generally available at 48-72 hours, are a major value for re-evaluation and reassessment of empirical therapy [28]. Though evidence from literature highlights the significance of de-escalation of antibiotics based on microbiological data, if patient's condition deteriorates or fails to improve by 48-72 hours, a careful re-evaluation and the possibility of infection with a resistant pathogen or a non-bacterial pathogen is considered [47]. Resource challenges and benefits from laboratory were lacking in our setting, hence “hit hard” strategy was being adopted in the critically ill patients with empirical broad-spectrum antibiotics. Initial empirical antibiotics choice was based on the treating clinician's choice, availability of antibiotics in the hospital, severity of illness and on health-care associated or community-acquired infection. A precise sample size was a limitation to our study, where an extremely small proportion of the global burden of infections in the critically ill children was taken into account. But, in the absence of other local sources of antibiotic prescribing practices, we understand that this study would help ascertain gaps in our knowledge and practices towards antibiotic prescribing in our resource limited set-ups in critically ill children with septic shock. There is need of a local antibiotic prescription guideline on septic shock. Training of clinicians and healthcare workers on rational empirical antibiotic use and appropriate de-escalation in pediatric septic shock management is very crucial to improve outcome.

## Conclusion

All children received empiric antibiotics on admission and majority (88%) received it in the initial one-hour. Blood cultures were done in only a quarter of patients prior to antibiotic administration. De-escalation of empirical antibiotics was not well accomplished due to microbiological culture limitations. Monotherapy was the commonest choice of antibiotics. Antibiotics were changed in children with clinical deterioration at 24 and 48 hours.

### What is known about this topic

- Early and appropriate use of antibiotics is associated with better outcome in septic shock children.

### What this study adds

- Antibiotic use in septic shock needs to be rationalized in resource limited settings where blood cultures and other inflammatory markers are not available frequently;
- Guideline on antibiotic use needs to be added to local paediatric guidelines to rationalize antibiotic use, improve outcome and reduce resistance.

## Competing interests

The authors declare no competing interests.
